# Cognitive control in number processing: new evidence from task switching

**DOI:** 10.1007/s00426-020-01418-w

**Published:** 2020-09-26

**Authors:** Andreas Schliephake, J. Bahnmueller, K. Willmes, K. Moeller

**Affiliations:** 1 Leibniz-Institut fuer Wissensmedien, Schleichstr. 6, 72076 Tübingen, Germany; 2 Centre for Mathematical Cognition, Loughborough University, Loughborough, LE11 3TU UK; 3 Department of Neurology, University Hospital, RWTH Aachen University, 52062 Aachen, Germany; 4 Department of Psychology and LEAD Graduate School & Research Network, University of Tübingen, 72076 Tübingen, Germany

## Abstract

Recently, it was demonstrated that even basic numerical cognition such as the processing of number magnitude is under cognitive control. However, evidence so far primarily came from adaptation effects to stimulus characteristics (e.g., relative frequency of specific stimulus categories). Expanding this approach, we evaluated a possible influence of more active exertion of cognitive control on basic number processing in task switching. Participants had to perform a magnitude comparison task while we manipulated the order of compatible and incompatible input–output modalities (i.e., auditory/vocal input–visual/manual output vs. auditory/visual input–manual/vocal output, respectively) on the trial level, differentiating repeat vs. switch trials. Results indicated that the numerical distance effect but not the problem size effect was increased after a switch in input–output modality compatibility. In sum, these findings substantiate that basic number processing is under cognitive control by providing first evidence that it is influenced by the active exertion of cognitive control as required in task switching.

## Introduction

In recent years, the impact of domain-general cognitive abilities on numerical cognition has gained increasing research interest (e.g., Cowan & Powell, 2014; Geary, 2011; Hohol, Cipora, Willmes, & Nuerk, 2017; Passolunghi and Lanfranchi, 2012; Peng, Namkung, Barnes, & Sun, 2016). One focus was on the influence of cognitive control designating the ability of the human cognitive system “to configure itself for the performance of specific tasks through appropriate adjustments in perceptual selection, response biasing, and the on-line maintenance of contextual information (Botvinick, Braver, Barch, Carter, & Cohen, 2001, p. 624)”. However, so far an influence of cognitive control on numerical cognition was primarily investigated when evaluating rather passive adaptation to stimulus set characteristics (Huber et al., 2013; Huber, Moeller, & Nuerk, 2014; Macizo & Herrera, 2011; Moeller, Klein, & Nuerk, 2013) or order (Macizo & Herrera, 2013; Pfister, Schroeder, & Kunde, 2013). Evidence for an influence of more active instantiations of cognitive control on numerical cognition is still scarce. By active we refer to situations, in which participants have to actively exert cognitive control to coordinate actions for the task at hand as, for instance, required in task switching paradigms. This seems all the more relevant, because numbers are commonly used stimuli in task-switching paradigms (e.g., Koch & Allport, 2006; Sudevan & Taylor, 1987), and there is first evidence in the literature that task switching may influence the processing of numerical information (Wendt, Kiesel, Mathew, Luna-Rodriguez, & Jacobsen, 2013). Accordingly, this study employed a task switching paradigm requiring participants to actively coordinate their response actions instead of passively adapting to stimuli characteristics. We evaluated whether cognitive control demands (i.e., switches between input–output modality couples) influenced basic number magnitude processing as reflected by the numerical distance effect and the problem size effect. Such alterations of number magnitude processing would provide further evidence that numerical cognition is under cognitive control.

In the following, we will first review recent findings regarding influences of cognitive control on numerical cognition. We then introduce the key notions of task switching, input–output modality compatibility and the respective numerical effects to be investigated in the current study before describing its details.

## Cognitive control in number processing

So far, evidence that number processing is under cognitive control was primarily derived from results indicating that participants adapt to changing stimulus set characteristics as reflected by alterations of the unit-decade compatibility and/or numerical distance effect (e.g., Moeller et al., 2013). The unit-decade compatibility effect (Nuerk, Weger, & Willmes, 2001) in two-digit number magnitude comparison describes the finding that responses to unit decade compatible number pairs (e.g., 52_67, both 5 < 6 and 2 < 7) are usually faster and less error prone than responses to unit decade incompatible pairs of matched overall distance (e.g., 58_73, 5 < 7 but 8 > 4). It was repeatedly observed that the size of the unit-decade compatibility effect depends on stimulus set characteristics. For instance, Huber et al. (2013) found that the size of the compatibility effect was associated with the percentage of within-decade filler items (e.g., 53_58) in the respective stimulus set. In particular, they observed the unit-decade compatibility effect to increase with the percentage of within-decade fillers. This finding reflects that with more within-decade filler items in the stimulus set, the unit digits become increasingly decision relevant. As the compatibility effect is a unit-based interference effect, this, in turn, increases the unit-decade compatibility effect. Huber et al. (2013) substantiated this argument in an eye-tracking study by observing that with increasing percentages of within-decade filler items participants fixated the unit digits more prominently—indicating their increased decision relevance. As such, this indicates that number processing is adapted to stimulus set characteristics.

Additional evidence for this claim comes from studies indicating that the unit-decade compatibility effect is not only influenced by the percentage of within-decade filler items, but also by the proportion and the sequence of compatible and incompatible trials. Macizo and Herrera (2011) found that the more incompatible trials were presented, the smaller was the unit decade compatibility effect because participants seemed to adapt to the prevalent task characteristics. This argument was further corroborated by the observation that the unit-decade compatibility effect was reduced when a compatible trial followed-up on two incompatible ones as compared to an incompatible trial followed-up on two compatible ones (Macizo & Herrera, 2013). Finally, this claim is supported by evidence from computational modelling (Huber et al., 2013; Huber, Nuerk, Willmes, & Moeller, 2016). In Huber et al. (2016) model of multi-digit number magnitude comparison, the respective modulations of the unit-decade compatibility effect can be accounted for by a cognitive control node adapting to the decision relevance of units, tens, hundreds, and so on. All these findings provide converging evidence for the argument that number processing is under cognitive control.

In line with these empirical findings, Hohol et al. (2017) recently suggested to investigate the role of cognitive control in numerical processing more systematically. However, to the best of our knowledge, previous studies almost exclusively evaluated influences of cognitive control on number processing as passive adaption to changing stimulus set characteristics (e.g., Huber et al. 2013) or order (e.g., Pfister et al., 2013). Therefore, we aimed at investigating how cognitive control affects basic numerical effects using a task switching paradigm, requiring the active exertion of cognitive control to adjust to task demands.

## Task switching

In task switching, one is required to shift between two or more different tasks. Switching from one task to another requires the cognitive system to change the respective mental task sets (for reviews, see Kiesel et al., 2010; Koch et al., 2010; Meiran, 2010). Accordingly, performance differences between switch and non-switch conditions are called switch costs, typically affecting both processing speed and accuracy (Huestegge, 2011; Pashler, 2000). Switch costs, to only name a few contexts/conditions, may arise from stimulus changes, task switches, response-mapping difficulty (Schumacher, Elston, & D’Esposito, 2003), stimulus modality incompatibility (e.g., Lukas, Philipp, & Koch, 2009), changes of response modality (e.g., Philipp & Koch, 2005), and input–output modality compatibility (Stephan & Koch, 2010, 2011).

Some theories accounting for task switching effects suggest a top-down controlled view assuming that switch costs reflect intentional control processes to actively reconfigure the mental task set (Monsell, 2003; Rogers & Monsell, 1995; Rubinstein, Meyer, & Evans, 2001). However, the fact that switch costs remain even when sufficient time for complete task set reconfiguration is provided questions the validity of theories claiming top-down control. Another influential switch costs theory emphasizes the inhibitory role of irrelevant task sets. Inhibitory processes may be triggered by conflict during several processing stages, particularly at the response selection stage (e.g., Koch et al., 2010; Schuch & Koch, 2003). In sum, it seems evident that intentional and automatic mechanisms are contributing to switch costs (e.g., Kiesel et al., 2010; Koch et al., 2010; Meiran, 2010; Monsell, 2003; Ruthruff, Remington, & Johnston, 2001).

Considering aforementioned theories from a cognitive control perspective, it seems evident that active exertion of cognitive control is necessary to perform in task switching situations. Moreover, as Kiesel et al. (2010) stated “the task-switching paradigm offers enormous possibilities to study cognitive control as well as task interference” (p.1). Accordingly, we chose a task switching paradigm to further evaluate how cognitive control influences numerical cognition. Regarding influences of task switching on number processing, Wendt et al. (2013) provided evidence for an influence of task switching on number processing. In Experiment 2 of their study, participants had to classify numbers according to their parity as odd or even and letters according to whether they were vowels or consonants. For the numerical stimuli, the size of the SNARC (Spatial-Numerical Association of Response Codes) effect was compared between repetition and switch trials. The authors observed that the SNARC effect (i.e., faster responses to relatively smaller numbers with the left and faster responses to relatively larger numbers with the right hand; see Dehaene, Bossini, & Giraux, 1993) was reduced in switch trials. Their explanation for the reduced SNARC effect was similar to that of Pfister et al. (2013), suggesting that the smaller SNARC effect might be accounted for by additional control processes required in a switch trial due to increased between-task interference. Thus, these results suggest that increasing cognitive control demands seem to reduce spatial-numerical associations as reflected by the SNARC effect.

Paradigms working with input–output modality changes represent one specific way to investigate task switching effects on numerical processing. So far, effects of input and output modality changes have mostly been investigated using dual-task experiments. Findings suggest that compatible modality pairs are processed faster than incompatible processing pathways. According to Hazeltine, Ruthruff, & Remington (2006), input–output modality combinations can be either compatible (visual input–manual output or auditory input–vocal output) or incompatible (visual input–vocal output or auditory input–manual output) due to “natural tendencies” of responding to visual stimuli manually and to auditory stimuli vocally. For example, Stephan and Koch (2010, 2011) specifically explored input–output modality compatibility effects in task switching. In that paradigm, participants were presented either auditory (i.e., 400 Hz tones on either the left or right ear via headphones) or visual stimuli (i.e., white diamonds on either the left or right of an otherwise black screen). Their task was to indicate whether a tone or a diamond was presented on the left or the right side. Responses had to be given either vocally by saying the word “left” or “right” or manually by pressing a left or right response key. In input–output compatible trials, auditory stimuli required a vocal response and visual stimuli required a manual response. Contrarily, in input–output incompatible trials, auditory stimuli required a manual response and visual stimuli required a vocal response. Modality switch costs were assessed for switches between input–output compatible tasks (auditory–vocal & visual–manual) and input–output incompatible tasks (auditory–manual & visual–vocal), respectively. No cue was necessary to indicate modality task switches because input–output pairings remained the same throughout the respective switch block (e.g., in the input–output compatible switch block auditory stimuli always required a vocal response and visual stimuli always required a manual response). Stephan and Koch (2010) found faster RT for modality compatible compared to modality incompatible response pairings. Crucially, modality switch costs were smaller for compatible input–output combinations than for incompatible input–output combinations. This indicates that cognitive control processes are required to compensate for the increased cross talk in incompatible modality pairs. Therefore, using an input–output modality compatibility paradigm with numerical stimuli seems suitable to investigate whether numerical effects (such as the numerical distance effect and problem size effect) are influenced by the active exertion of cognitive control. In the next section we provide a brief description of these two effects of interest.

## Numerical distance and problem size effect

Numerical magnitude is the primary information to be represented by numbers. Several behavioural effects allow for inferences about the mental representation of number magnitude (Dehaene, 1992; Dehaene et al., 1993; Fias, 1996). For instance, the numerical distance effect (Moyer & Landauer, 1967) describes the negative relationship between the absolute numerical distance between two numbers and the time needed to compare the two numbers regarding their magnitude. In other words, it reflects that comparing numerically closer numbers (e.g., 4 and 5) is slower and more error prone than comparing more distant ones (e.g., 1 and 9). Usually, the numerical distance effect is accounted for by the assumption that mental representations of closer numbers overlap more than those of more distant numbers, which makes the latter easier to distinguish. Moreover, the problem size effect reflects that performance with numbers decreases as their magnitude increases. For instance, when distance is kept constant comparisons of two numbers are more difficult (in terms of longer RT and more errors for larger numbers (e.g., 517 vs. 519) as compared to smaller ones (17 vs. 19, e.g., Brysbaert, 1995; Parkman, 1971).

This is of particular relevance, as these effects reflect basic number magnitude processing. As such, in case we find alterations to these numerical effects in a task switching paradigm, this would indicate an influence of the active exertion of cognitive control on basic number processing.

## The present study

In the current study, we adapted the input–output modality switch paradigm by Stephan and Koch (2010) employing numerical stimuli. Adding to previous studies on cognitive control in numerical cognition, we specifically evaluated whether the active exertion of cognitive control in task switching altered the numerical distance effect and/or problem size effect, both of these hallmark effects reflecting core number magnitude processing. In particular, differing from Stephan and Koch (2010), participants had to decide on each trial whether a presented single-digit number was smaller or larger than the reference value 5. Because of differential modality switch costs for input–output compatible and incompatible combinations reported by Stephan and Koch (2010), we kept the input–output compatibility manipulation. In two switch blocks participants switched between compatible modality pairs (auditory–vocal & visual–manual) or between incompatible modality pairs (auditory–manual & visual–vocal), respectively. No cue indicating modality task switches was necessary in our study as well because input–output pairings remained the same throughout the respective switch block (e.g., in the input–output compatible switch block hearing a single-digit number required a vocal response and seeing a single-digit number always required a manual response; i.e., “left” for smaller than five, “right” for larger than five, respectively).

First, we expected to replicate the input–output modality compatibility effect (Stephan and Koch, 2010) as well as its interaction with task switching (i.e., lager switch costs in input–output incompatible compared to input–output compatible combinations) as a baseline. Second, we expected to observe the problem size effect and the numerical distance effect to be modulated by input–output modality task switching. In line with preliminary observations of influences of task switching on number processing (e.g., Wendt et al., 2013), we predicted the numerical distance and problem size effect to be smaller in switch trials as compared to repeat trials.

## Methods

### Participants

A total of *n* = 32 participants was tested (10 men, mean age = 25.3 years, SD = 5.7 years). Hearing and visual acuity were normal or corrected to normal. All participants were students (90.6%) or academics. Participation was voluntary and compensated with 10 €. The study was conducted in accordance with the latest version of the Helsinki convention.

### Task and materials

Single-digit numbers from 1 to 9 except for 5 served as stimuli and were presented either in the auditory or the visual modality. Going beyond previous studies, we controlled for interference by previous stimuli caused by the rapid succession of stimuli (e.g., Neely, 1977). According to Nuerk, Wood, and Willmes (2005) for number stimuli this means that processing the magnitude of the number in the previous trial might interfere with processing number magnitude in the current trial. Therefore, we balanced the overall frequency of the number stimuli in each block (i.e., each number was presented equally often) and the frequency of all possible transitions between two numbers to ensure that observed effects can be attributed to the active exertion of cognitive control called for in task switching.

Auditory stimuli were number words spoken in German. These were presented through binaural headsets (Speed Link SL-8755) at a frequency of 400 Hz. Visual stimuli were Arabic digits. They were displayed with a size of 2.4 cm in white on a black background screen at the centre of a 19-in. computer monitor. Viewing distance was approximately 60 cm. Responses were given vocally or manually. Vocal responses were the German words for left (“links”) indicating that the number is smaller than five and right (“rechts”) indicating that the presented number is larger than five. A voice key registered speech onset as RT and the experimenter coded the response online via the keyboard’s number pad with the “Enter” or the “+” key respectively. In manual trials the left (“Ctrl”) or right key (“Alt”) of a QWERTZ keyboard had to be pressed with the index finger of the left or right hand, respectively. The experiment was programmed using MATLAB’s Brainard-Pelli Psychophysics Toolbox.

### Procedure

Participants were instructed to categorize single-digit numbers as smaller or larger than five, while being as accurate and fast as possible. Each participant took part in the single-task and the task-switch condition, see Table 1 for an illustration. The order of both conditions was counterbalanced across participants.

**Table 1:**
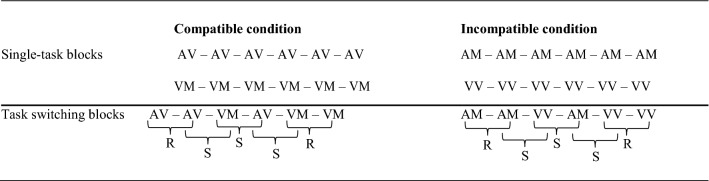
Scheme of the experimental design reflecting sequences of compatible and incompatible input-output modality pairings (i.e., items) in single-task blocks and task switching blocks

Please note that trial order in task switching blocks was pseudo-random. *AV* auditory-vocal, *VM* visual-manual, *AM* auditory-manual, *VV* visual-vocal, *R* repetition trial, *S* switch trial

In the single-task condition, a total of four single-task blocks was administered. Two blocks with compatible input–output pairings (auditory-vocal & visual-manual) and two blocks with incompatible input–output pairings (auditory-manual & visual-vocal). Each single-task block had two introductory trials, 4 practice trials, and 56 experimental trials. Thus, each number was presented seven times in each single task block.

The task switching condition had two task switching blocks—one input–output compatible and one input–output incompatible block. Thus, in the compatible block, participants had to switch between auditory-vocal and visual-manual pairings. In the incompatible block, participants had to switch between auditory-manual and visual-vocal pairings. Switch trials had an identical frequency of modality pairings and all possible transitions between two number stimuli were counterbalanced. The switch condition started with 2 introductory followed by 8 practice trials, and 112 experimental trials. Thus, each number was presented 14 times in switch blocks. In switch trials no cue indicated modality task switches because input–output pairings remained the same throughout the respective switch block (e.g., in the input–output compatible switch block hearing a single-digit number required a vocal response and seeing a single-digit number always required a manual response). One session lasted about 40 min.

Pseudo-random trial sequences conforming to the above described constraints were generated prior to the experimental session. Every trial started with stimulus presentation and ended with the response or after a maximum response interval of 2500 ms. Intervals from stimulus onset to response onset were measured as RT. A correct response was followed by the start of the next trial after 600 ms (RSI, response-stimulus interval). In case of incorrect or missing responses an error message (i.e. the German word for error, “Fehler”) appeared on the screen for 500 ms extending the RSI to 1100 ms.

## Results

### Analysis

Before the analysis, incomplete data (either response key or response time not registered) were removed. The dependent variable of interest was RT. A fixed cut-off of RT < 50 ms was applied to eliminate premature responses or voice-key artefacts. In addition, RT outliers of ± 3 SD were also eliminated from the data set. Additionally, error trials and trials immediately following an error were discarded. This resulted in a loss of approximately 11.88% of the data. To control for input-specific or output-specific effects the data of compatible tasks and of incompatible tasks, respectively were merged for analysis (see Stephan and Koch, 2011). Data preparation and analyses were done in R.

In a first step, we investigated the presence of the numerical distance effect, the problem size effect and the modality compatibility effect by jointly considering data of all four single-task blocks. To do so we employed an ANOVA comprising the factors numerical distance reflecting the absolute distance of the numerical stimulus to 5 (small, i.e., 1, 2 vs. large, i.e., 3, 4), problem size reflecting the relative size of the number in the stimulus set (small < 5 vs. large > 5), and input–output modality compatibility (compatible vs. incompatible). As a second step and focusing on the task switching blocks, we then evaluated separately for the numerical distance and problem size effect whether effects of input–output modality task switching modulated the numerical effects. Therefore, we used within-participant ANOVAs employing the factors input–output modality compatibility (compatible vs. incompatible), task switching (repetition vs. switch) as well as numerical distance (small vs. large) or problem size (small vs. large). Significant interaction effects of the ANOVA were followed-up by paired sample *t*-tests to evaluate simple effects.

### Single-task blocks

We observed significant main effects of numerical distance [*F*(1, 31) = 103.41, *p* < .001; *η*_p_^2^ = .74], problem size [*F*(1, 31) = 11.02 *p* < .001; *η*_p_^2^ = .50] and input–output modality compatibility [*F*(1, 31) = 11.6, *p* < .001; *η*_p_^2^ = .57]. Mean RT were shorter for large numerical distances (*M* = 578 ms, SD = 85 ms) than for small numerical distances (*M* = 618 ms, SD = 92 ms); this relation was present in 31/32 individual participants (97%). Mean RT were also shorter for smaller problem size (*M* = 589 ms, SD = 87 ms) as compared to larger problem size (*M* = 607 ms, SD = 94 ms); this relation was present in 26/32 participants (81%). Finally, input–output modality compatible trials had shorter average RT (*M* = 568 ms, SD = 89 ms) as compared to incompatible trials (*M* = 627 ms, SD = 83 ms), this relation being present in 25/32 participants (78%). Additionally, a significant ordinal interaction between problem size and numerical distance was found [*F*(1, 31) = 6.20, *p* = .02; *η*_p_^2^ = .50]: the numerical distance effect was more pronounced in trials with large as compared to small problem size (40 ms vs. 24 ms, respectively).

### Modulation of the numerical distance effect by task switching

There was a significant main effect of input–output modality compatibility on RT [*F*(1, 31) = 36.21, *p* < .001; *η*_p_^2^ = .54]: mean RT was shorter in the compatible condition (*M* = 716 ms, SD = 114 ms) compared to the incompatible condition (*M* = 800 ms, SD = 172 ms). Furthermore, the respective incompatibility costs were positive in 28/32 individual participants (88%). Task switching (repetition vs. switch) also had a significant main effect on RT [*F*(1, 31) = 72.10, *p *<.001; *η*_p_^2^ = .70] with faster reaction times in repetition trials (*M* = 711 ms, SD = 113 ms) than in switch trials (*M* = 806 ms, SD = 170 ms). Switch costs were positive in 31/32 participants (97%). Moreover, the main effect of numerical distance on RT was significant as well [*F*(1,31) = 188.67, *p *<.001; *η*_p_^2^ = .86]. Reaction times were faster for large (*M* = 736 ms, SD = 147 ms) as compared to small numerical distances (*M* = 781 ms, SD = 153 ms). All participants showed longer reaction times for small as compared to large numerical distances, reflecting the numerical distance effect.

The interaction between task switching and numerical distance was also significant [*F*(1, 31) = 7.42, *p* = .01; *η*_p_^2^ = .19]. The numerical distance effect was larger in switch trials (*M* = 53 ms, SD = 28 ms) compared to repetition trials (*M* = 37 ms, SD = 21 ms). Furthermore, numerical distance effects were positive in all participants for repetition trials and 30/32 participants (94%) in switch trials. To assess whether a significant main effect of numerical distance was present in repetition as well as switch trials, simple effects were tested for. Simple effects indicated that the numerical distance effect was significant in both switch [*F*(1, 31) = 98,10, *p* < .001; *η*_p_^2^ = .76] and repetition trials [*F*(1, 31) = 112.92, *p* < .001; *η*_p_^2^ = .78].

Moreover, the interaction between input–output modality compatibility and task switching [*F*(1, 31) = 47.76, *p* < .001; *η*_p_^2^ = .61] was significant. Replicating results by Stephan and Koch (2010), switch costs were larger in the input–output modality incompatible (*M* = 141 ms, SD = 92 ms) than in the modality compatible condition (*M* = 50 ms, SD = 48 ms). Switch costs were positive for 28/32 individual participants (88%) in the modality compatible condition and positive for all participants in the input–output modality incompatible condition.

**Fig. 1:**
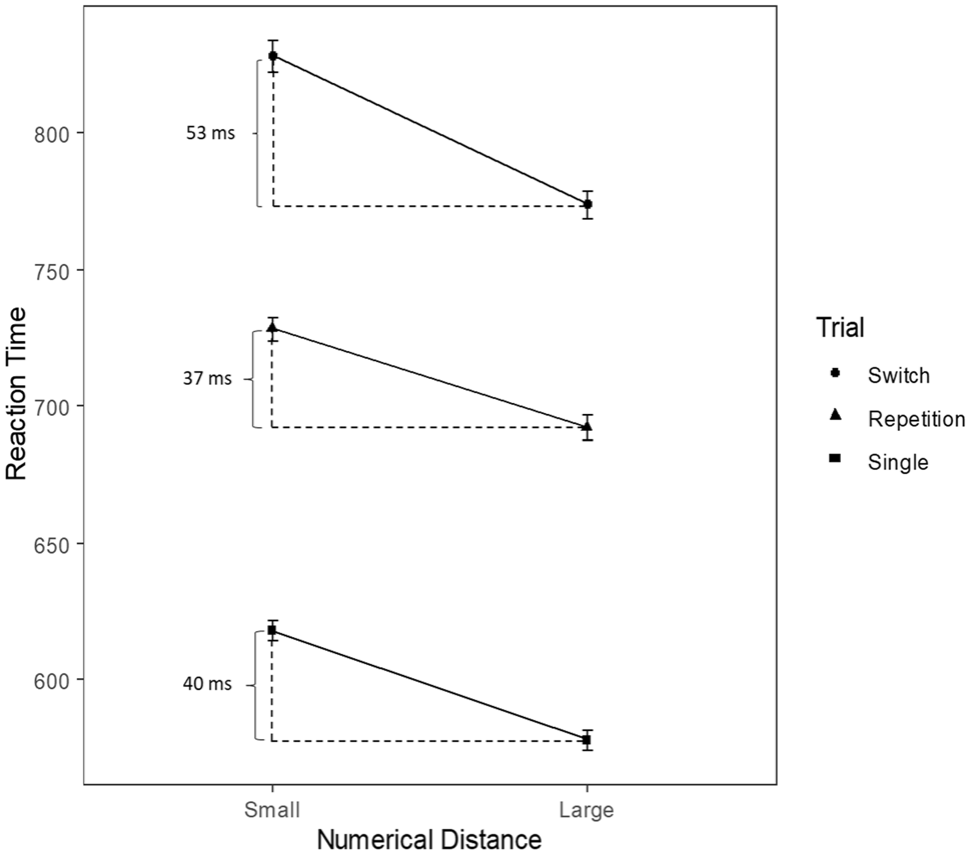
Illustration of the numerical distance effect for single-task (mean difference between numerical distance effects 40 ms), repetition (37 ms) and switch (53 ms) trials. Error bars indicate 1 standard error of the mean (SEM)

Finally, the numerical distance effect did not differ significantly between the single-task condition (*M* = 40 ms, SD = 23 ms) and the repetition trials (*M* = 37 ms, SD = 21 ms) of the task-switch blocks [*t*(31) = 0.49, *p* = .63]. However, the numerical distance effect differed significantly in switch trials of the task-switch condition compared to the repetition trials of the task-switch condition [*t*(31) = 2.70, *p* = .01] with switch trials showing larger numerical distance effects (*M* = 53 ms, SD = 28 ms) (Fig. 1).

### Modulation of the problem size effect by task switching

Generally, we observed the same main effects for input–output modality compatibility [*F*(1, 31) = 36.21, *p* < .001; *η*_p_^2^ = .54] and task switching [*F*(1, 31) = 72.10, *p *<.001; *η*_p_^2^ = .70] as well as their interaction [*F*(1, 31) = 47.76, *p* < .001; *η*_p_^2^ = .61] as in the ANOVA evaluating the numerical distance effect and its modulation by input–output modality compatibility and task switching. Replicating results by Stephan and Koch (2010), switch costs were larger in the input–output modality incompatible (*M* = 141 ms, SD = 92 ms) than in the modality compatible condition (*M* = 50 ms, SD = 48 ms). This reflected that mean RT was shorter in the compatible condition (*M* = 716 ms, SD = 114 ms) compared to the incompatible condition (*M* = 800 ms, SD = 172 ms). Furthermore, the respective incompatibility costs were positive in 28/32 individual participants (88%). Moreover, reaction times were faster in repetition trials (*M* = 711 ms, SD = 113 ms) than in switch trials (*M* = 806 ms, SD = 170 ms). The respective switch costs were positive in 31/32 participants (97%). Switch costs were positive for 19/32 individual participants (59.4%) in the modality compatible condition and positive for 24/32 participants (66.7%) in the modality incompatible condition.

Additionally, the main effect of problem size on RT was significant as well [*F*(1, 31) = 11.30, *p *<.01; *η*_p_^2^ = .27]. Reaction times were faster for smaller numbers (*M* = 753 ms, SD = 149 ms) as compared to larger numbers (*M* = 764 ms, SD = 152 ms). Overall, 24/32 participants (75%) showed smaller reaction times for smaller as compared to larger numbers reflecting the problem size effect.

**Fig. 2:**
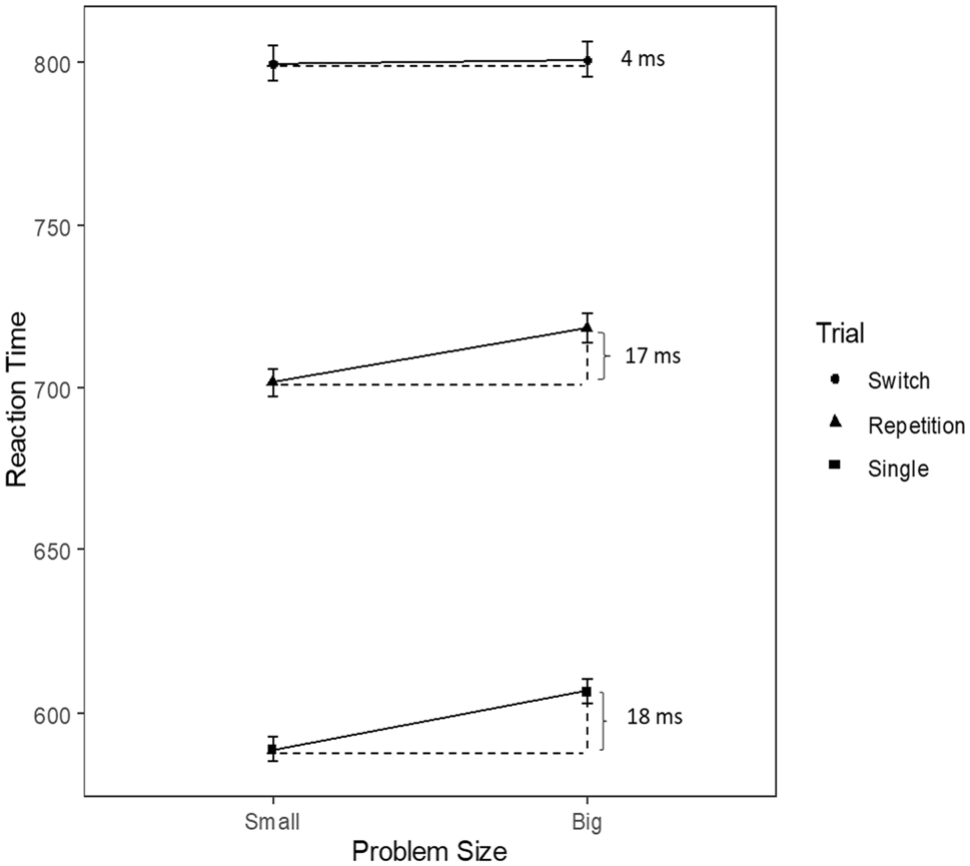
Illustration of the problem size effect for single-task (mean difference between problem size effects 18 ms), repetition (17 ms) and switch (4 ms) trials. Error bars indicate 1 SEM

The interaction between problem size and task switching was not significant [*F*(1, 31) = 2.25, *p* = .14; *η*_p_^2^ = .07]. The problem size effect was not significantly different between single-task condition (*M* = 18 ms, SD = 18 ms), repetition (*M* = 17 ms, SD = 29 ms), and switch trials (*M* = 4 ms, SD = 31 ms) (Fig. 2).

## Discussion

In this study we evaluated how task switching, requiring active exertion of cognitive control, influences basic number processing (as reflected by the numerical distance and problem size effect) using an input–output modality task switching paradigm (Stephan and Koch, 2011) with numerical stimuli. The paradigm required both, a numerical magnitude comparison and matching of output to input modality. Participants had to categorise single-digit numbers as smaller or larger than five while adjusting to pseudo-randomly varied input–output modality pairings. In line with our expectations, we replicated the input–output modality compatibility effect and its modulation through task switching. This extends the observation of these effects from simple perception-reaction pairings in Stephan and Koch (2010, i.e., location of stimuli indicating response modality) to the processing of semantic numerical knowledge.

Most importantly, however, we observed a modulation of the numerical distance effect—but not the problem size effect—by task switching. In the following we elaborate on how our findings regarding the modulation of the numerical distance effect add to the understanding of influences of cognitive control on number processing by providing first evidence on effects of active exertion of cognitive control.

We observed the numerical distance effect to be larger in switch trials compared to repetition trials. In terms of the additive factors method (Sternberg, 2011), this indicates that the cognitive processing modules involved were affected by both switching between input–output modality pairings and number magnitude comparison. In other words, the need to actively exert cognitive control in task switching situations affected the processing of number magnitude information underlying the numerical distance effect.

So far, findings regarding input–output modality compatibility, dual tasking (e.g., Hazeltine et al., 2006) and task switching (Stephan and Koch, 2010, 2011) point towards the response selection stage as the source of these effects. This inference is in line with further literature on human information processing, as incompatibilities between stimuli and responses of various nature were shown to influence response selection (Cohen Kadosh, Gevers, & Notebaert, 2011; Proctor & Vu, 2006; Sanders, 1998; Sternberg, 1998). In this regard, a plausible explanation for the increased numerical distance effect in switch trials may be the increased cross-talk during task switching. The term cross-talk is adopted from dual-task research and describes the overlap and resulting interference of content-dependent codes across tasks (Hazeltine et al., 2006; Koch, 2009). Considering the findings of our study, this means that the increase of the numerical distance effect in switch trials may originate from the overlap of number magnitude processing and the cognitively controlled activation and/or selection of the respective output modality. Such an explanation is supported by theories that relate the numerical distance effect to overlapping activations of adjacent numbers on the mental number line (e.g., Dehaene, 1992; Gallistel & Gelman, 1992) and is supported by general evidence of the impact of top-down control on bottom-up processing such as the automatic magnitude processing of small numbers (e.g., Cohen Kadosh, Bien, & Sack, 2012; Van Opstal, de Lange, & Dehaene, 2011).

As the numerical distance effect was accentuated during the prolonged decisional process of matching the changing input–output modalities, we suggest that the prolonged decisional process may reflect concurrent processing demands due to the simultaneous semantic processing of numbers and input–output modality changes. It seems obvious that these concurrent processing demands may lead to reduced processing efficiency. As such, these data provide converging evidence that number magnitude processing is under cognitive control—not only as reflected by effects of passive adaptation to stimulus set characteristics (e.g., Huber et al., 2013), but also in situations requiring the active exertion of cognitive control.

Notably, the problem size effect was not affected significantly by task switching. To account for this finding different theories regarding the origins of both numerical effects may be considered in the context of task switching. Regarding the numerical distance effect scientists argue that it reflects an underlying spatial representation of number magnitude along a mental number line (e.g. Dehaene, 1992). While the metaphor of the mental number line may also account for the problem size effect (e.g., Pinhas, Tzelgov, & Guata-Yaakobi, 2010), theories on the problem size effect usually emphasize more familiarity with smaller numbers to be at its origin (Dehaene, 1992; Gallistel & Gelman, 1992). For instance, Dehaene and Mehler (1992) proposed that the more frequent occurrence of small numbers in daily life would increase the familiarity with small numbers and thereby induce the problem size effect. Furthermore, processing smaller numbers is commonly assumed to be overlearned and therefore the magnitude comparison may occur even more automatically, prompted by the strength of pre-existing associations between number representations and response components (Cohen Kadosh et al., 2012; Van Opstal et al., 2011). Consequently, on a theoretical level one might argue that the numerical distance effect might have a different origin than the problem size effect. Moreover, as only single-digit numbers were used as stimuli in our study, a replication with larger two-digit numbers may allow for evaluating whether the small overall problem size was the reason that no effect of cognitive control on the problem size effect was found.

In sum, the present study provides converging evidence that even basic numerical cognition such as the processing of number magnitude is under cognitive control. Adding to previous studies primarily reporting evidence from adaptation effects to stimulus characteristics, we evaluated the influence of active exertion of cognitive control on basic number processing by employing a task switching paradigm. Participants performed a magnitude comparison task while we manipulated the order of compatible and incompatible input–output modalities (i.e., auditory/visual input–vocal/manual output vs. auditory/visual input–manual/vocal output, respectively) on the trial level differentiating repeat vs. switch trials. Our results indicated that the numerical distance effect but not the problem size effect was elevated after a switch in input–output modality compatibility. This finding further substantiates that basic number processing is influenced by the active exertion of cognitive control as required in task switching situations. Furthermore, on a conceptual level our findings may allow to differentiate potential origins of numerical effects. Thus, further studies investigating the interplay between active cognitive control exertion and numerical effects may not only have the potential to elucidate the role of cognitive control in number processing but might also help to better understand standard effects of number processing.

## Data Availability

The datasets of the current study are available from the corresponding author on reasonable request.
